# A Clinical and Molecular Comparative Analysis of KRAS Exon 2 and KRAS Non-Exon 2 Mutated Colorectal Cancer

**DOI:** 10.3390/cancers18132158

**Published:** 2026-07-05

**Authors:** Doga Kahramangil Baytar, Paola Zinser-Peniche, Shuaichao Wang, Yu Jen Alexander Jan, Ashley McFarquhar, Aatur Singhi, Anwaar Saeed, Ibrahim Halil Sahin

**Affiliations:** 1 Department of Medicine, University of Pittsburgh Medical Center (UPMC), Pittsburgh, PA 15213, USA; kahramangilbaytad@upmc.edu (D.K.B.);; 2 UPMC Hillman Cancer Center, Pittsburgh, PA 15213, USA; 3 Division of Hematology & Oncology, University of Pittsburgh Medical Center (UPMC), Pittsburgh, PA 15213, USA; 4 Department of Pathology, University of Pittsburgh Medical Center (UPMC), Pittsburgh, PA 15213, USA; 5 Division of Hematology & Oncology, University of Michigan Medical School, Ann Arbor, MI 48109, USA

**Keywords:** colorectal cancer, KRAS mutation, KRAS mutation subtypes, KRAS exon 2, KRAS non-exon 2, inflammatory markers, neutrophil-to-lymphocyte ratio

## Abstract

Background: Mutations in the KRAS oncogene occur in approximately 40% of colorectal cancers, predominantly within exon 2. Non-exon 2 mutations are less common and remain poorly characterized in terms of their clinical and biological significance. Systemic inflammatory markers are well-established prognostic indicators in colorectal cancer, yet whether their prognostic value differs across KRAS mutation subtypes has yet to be defined. We aimed to characterize and compare the clinicopathological and inflammatory profiles of patients with exon 2 versus non-exon 2 KRAS-mutated colorectal cancer and evaluate their prognostic implications. Methods: This retrospective cohort study analyzed 272 patients with microsatellite stable metastatic colorectal cancer with KRAS mutations, comprising 236 exon 2 and 36 non-exon 2 cases. Clinical, molecular, and laboratory data, including baseline systemic inflammatory markers, were extracted from electronic medical records. Survival outcomes and the prognostic impact of these variables were evaluated with Kaplan-Meier curves and univariable and multivariable Cox proportional hazards regression analyses. Results: Non-exon 2 mutations were significantly more frequent in female patients (64% vs. 45%, *p* = 0.048) and in left-sided primary tumors (83% vs. 64%, *p* = 0.035). Median overall survival was 45.7 months for the non-exon 2 group compared to 32.4 months for the exon 2 cohort; KRAS mutation subtype was not significantly associated with overall survival on univariable or multivariable analysis (univariable HR 1.36, 95% CI 0.85–2.16, *p* = 0.2; multivariable HR 1.376, 95% CI 0.794–2.383, *p* = 0.255). Systemic inflammation demonstrated distinct prognostic value, with elevated white blood cells, absolute neutrophil count, platelets, neutrophil-to-lymphocyte ratio, platelet-to-lymphocyte ratio, and low albumin levels demonstrating association with worse overall survival in the exon 2 cohort. Conversely, only an elevated neutrophil-to-lymphocyte ratio predicted worse survival in the non-exon 2 group. Conclusions: KRAS exon 2 and non-exon 2 mutated metastatic colorectal cancers exhibit distinct clinical and inflammatory characteristics. Systemic inflammation exerts a significantly greater prognostic impact in exon 2 disease. As the therapeutic landscape for KRAS-mutated CRC continues to evolve, these findings hold promise for informing KRAS mutation-specific approaches to patient stratification and treatment planning.

## Simple Summary

Mutations in the KRAS gene are among the most frequent drivers of colorectal cancer (CRC), and KRAS-mutated CRC is increasingly recognized as a heterogeneous disease. KRAS gene contains multiple coding regions, exons, which when mutated give rise to distinct tumor subtypes. Clinical and biological differences between the prevalent exon 2 mutations and the less common non-exon 2 mutations remain underexplored, including their effects on systemic inflammation and patient survival. In this study, we examined clinical characteristics and inflammatory markers across these subtypes to identify features with prognostic value. Elevated inflammatory markers predicted worse survival in patients with exon 2 mutations, while their prognostic impact was largely absent in those with non-exon 2 mutations. These findings highlight meaningful biological differences between KRAS mutation subtypes and support the need for larger studies as the treatment landscape for KRAS-mutated CRC continues to evolve.

## 1. Introduction

Colorectal cancer (CRC) is the third most common cancer by incidence and the second leading cause of cancer-related deaths, with an estimated 1.9 million new cases and 935,000 deaths annually, accounting for approximately one in ten cancer diagnoses and deaths worldwide [1]. Advances in precision oncology have transformed the management of CRC, enabling molecular profiling of tumors that facilitates the identification of actionable mutations to guide treatment decisions. Kirsten rat sarcoma (KRAS) gene is among the most frequently mutated oncogenes in CRC, with mutations found in approximately 40% of CRC cases. It encodes a small GTPase that acts as a downstream mediator of epidermal growth factor receptor (EGFR) signaling and regulates key cellular processes involved in carcinogenesis, including cell proliferation, differentiation, and survival [2]. Oncogenic KRAS mutations lock the protein in a constitutively active state, driving downstream signaling independent of upstream EGFR activation. This renders KRAS mutated tumors resistant to anti-EGFR therapies, establishing KRAS mutation as a negative biomarker for anti-EGFR treatment [3]. Given the high mutation rate and treatment implications, KRAS mutational testing becomes central in treatment planning.

The KRAS gene consists of six exons, with the vast majority of activating mutations occurring in exon 2 at codons 12 and 13, accounting for approximately 90% of all KRAS mutations in CRC [2,4]. This is followed by exon 3 mutations harboring codon 61, accounting for approximately 2-3% of KRAS mutations in CRC, while exon 4 contains codons 117 and 146, affected in roughly 3–4% of cases [5].Our understanding of KRAS biology has been mostly shaped by exon 2 mutations, both due to their biological dominance and high prevalence and the development of targeted agents such as sotorasib and adagrasib, which specifically inhibit the G12C mutant protein of codon 12 [3]. Historically, clinical testing focused predominantly on KRAS exon 2 mutations; as extended RAS analyses demonstrated that non-exon 2 mutations similarly predict resistance to anti-EGFR therapy, broader mutational testing has become standard practice [3,6]. Despite this, the clinical and prognostic significance of non-exon 2 mutations remains relatively underexplored.

Systemic inflammatory markers have emerged as prognostic biomarkers in CRC, with elevated neutrophil-to-lymphocyte ratio (NLR) and platelet-to-lymphocyte ratio (PLR) being associated with inferior survival outcomes [7,8]. Growing evidence also implicates KRAS mutation as a modulator of the tumor immune microenvironment, with distinct patterns of immune infiltration reported across different KRAS mutation subtypes [9]. Despite this biological link, the prognostic implications of these systemic inflammatory markers have not been characterized in the context of different KRAS mutation subtypes.

With this study, we aimed to characterize and compare the clinicopathological, molecular, and inflammatory features of patients with exon 2 versus non-exon 2 KRAS-mutated CRC, and to evaluate the prognostic impact of these characteristics on survival outcomes.

## 2. Materials and Methods

### 2.1. Study Design and Patient Selection

We conducted a retrospective, observational cohort study at UPMC Hillman Cancer Center. Patients aged 18 years or older with metastatic CRC (mCRC) who underwent genomic profiling using Oncomine Next-Generation Sequencing (NGS) at our institution between 2019 and 2025 were screened for eligibility. Patients were included if their tumor demonstrated either a KRAS exon 2 or KRAS non-exon 2 mutation. Patients with non-metastatic (non-stage IV) disease, microsatellite instability-high (MSI-H) tumors, or insufficient molecular data were excluded. For patients where both exon 2 and non-exon 2 KRAS mutations were identified on next-generation sequencing, cases were included in the exon 2 mutated group. Evidence suggests that codon 12 mutations more completely impair GTPase activity and are associated with higher levels of constitutively active GTP-bound KRAS compared to non-exon 2 variants [10,11,12,13], suggesting that exon 2 mutations would likely dominate oncogenic MAPK signaling when co-occurring. Additionally, this classification was intended to preserve the biological purity of the non-exon 2 cohort, which is the primary group of interest in this study. A total of 236 patients with KRAS exon 2 mutations and 36 patients with KRAS non-exon 2 mutations met inclusion criteria and were analyzed. Survival data were collected through April 2026.

### 2.2. Data Collection

Demographic, clinical, laboratory, and molecular data were collected through retrospective review of electronic medical records. Demographic variables included age at diagnosis, sex, patient-reported race, and smoking status. Clinical variables included ECOG performance status at the time of stage IV disease diagnosis, primary tumor location (colon vs. rectum), tumor sidedness (right-sided vs. left-sided), delivery of first line systemic treatment, type of first line systemic treatment (oxaliplatin-based doublet, irinotecan -based doublet, triplet, fluoropyrimidine monotherapy, or other), and metastatic site involvement (lung, liver, peritoneum, other) and metastatic burden (number of anatomic sites involved by metastatic disease, single vs. multiple). Right-sided tumors were defined as those arising from cecum, ascending colon, hepatic flexure, or transverse colon; left-sided tumors included splenic flexure, descending colon, sigmoid colon, and rectum. Dates of first-line and second-line systemic treatment initiation were collected to estimate time to next treatment (TTNT). Date of death (DoD), or date of last contact where DoD was not available, were recorded to estimate overall survival (OS). Collected laboratory values were obtained at the time of stage IV disease diagnosis. These included WBC, absolute neutrophil and lymphocyte counts (ANC and ALC), platelet count, serum albumin, CEA, and CA19-9. Laboratory values were interpreted (low/normal vs. High) according to defined ranges per UPMC laboratory. Neutrophil-to-lymphocyte ratio (NLR) and platelet-to-lymphocyte (PLR) ratio were calculated from collected numbers. Molecular data were curated from pre-existing institutional database including KRAS exon subtype and mutation type, PIK3CA mutation status, and tumor mutational burden (TMB).

### 2.3. Statistical Analysis

Categorical variables were compared using Fisher’s Exact test. Continuous variables were summarized as medians with range and compared using the Mann-Whitney U test. OS was defined as the time from stage IV disease diagnosis to death from any cause or date of last contact. Time to next treatment (TTNT) was defined as the time from first-line to second-line systemic treatment initiation, or death from any cause. Patients without documented second-line treatment initiation or death were censored at the date of last contact. Patients without first-line treatment data were excluded from this analysis.

Survival distributions were compared between groups using the Kaplan-Meier method and log-rank test. Hazard ratios and 95% confidence intervals were estimated using univariable Cox proportional hazards regression analyses to assess the association of clinical characteristics and inflammatory markers with OS. Multivariable Cox proportional hazards regression was performed in the overall cohort, incorporating following covariates: KRAS mutation subtype, age at diagnosis, ECOG performance status, tumor sidedness, delivery of first-line systemic treatment, metastatic burden (single site vs. multiple metastatic sites), PIK3CA co-mutation status, PLR, NLR, and serum albumin. Patients with missing covariate data were excluded from multivariable analyses. A two-sided *p*-value of <0.05 was considered statistically significant.

## 3. Results

### 3.1. Patient Characteristics

Of the 272 patients with KRAS-mutated MSS mCRC included in this analysis, 36 (13%) had KRAS non-exon 2 mutations and 236 (87%) had KRAS exon 2 mutations. Baseline characteristics are summarized in Table 1. The median ages at diagnosis were 61.0 and 61.5 years in the non-exon 2 and exon 2 groups, respectively (*p* = 0.255). Non-exon 2 KRAS mutations were significantly more common among female patients compared to exon 2 mutations (64% vs. 45%; *p* = 0.048). Both non-exon 2 and exon 2 groups were predominantly of White race (non-exon 2: 89%; exon 2: 83%; *p* = 0.562). Smoking status (*p* = 0.461) and ECOG performance status (*p* = 0.120) were similar between two groups.

Although primary tumors were more commonly located in the colon, compared to rectum, in both groups (non-exon 2: 69%; exon 2: 72%; *p* = 0.694), notably, non-exon 2 mutations were significantly more frequently associated with left-sided primary tumors compared to exon 2 mutations (83% vs. 64%; *p* = 0.035). The number of metastatic sites did not differ significantly between groups, with single-site metastasis present in 56% of non-exon 2 and 61% of exon 2 patients (*p* = 0.584). The rate of liver, lung, peritoneal, and other metastases also did not differ significantly between groups (*p* > 0.05 in all comparisons).

Delivery of first-line systemic treatment was significantly higher in the non-exon 2 group compared to the exon 2 group (89% vs. 66%; *p* = 0.004). Among treated patients, irinotecan-based doublet regimens were more frequently used in the non-exon 2 group (36% vs. 14%), while oxaliplatin-based doublets were the most common regimen in both groups (42% vs. 41%). The overall distribution of first-line regimens differed significantly between groups (*p* = 0.003).

### 3.2. Molecular Characteristics

PIK3CA co-mutations were present in 8.3% of non-exon 2 and 11% of exon 2 patients (*p* = 0.778). Median TMB was 9.36 mutations/Mb in the non-exon 2 group and 8.86 mutations/Mb in the exon 2 group (*p* = 0.546). Serum CA19-9 (median 33 vs. 55.45; *p* = 0.395) and CEA (median 17.4 vs. 17.1; *p* = 0.797) levels did not differ significantly between the non-exon 2 and exon 2 groups.

### 3.3. Inflammatory Markers

Elevated platelet counts were significantly more common in the exon 2 group (5.6% vs. 28%; *p* = 0.008). Median WBC was significantly higher in the exon 2 compared to non-exon 2 group when analyzed as a continuous variable (7.9 vs 7.05 ×10^3^/μL; *p* = 0.032), though this significance was not present when analyzed categorically (*p* = 0.179). No significant differences were observed for NLR (median 4.31 vs. 3.91; *p* = 0.450), PLR (median 230.91 vs. 208.39; *p* = 0.430), ANC (*p* = 0.205), ALC (*p* = 0.071), or serum albumin (median 3.85 vs. 3.70 g/dL; *p* = 0.177) between non-exon 2 and exon 2 groups.

### 3.4. Survival Outcomes

**Figure 1:**
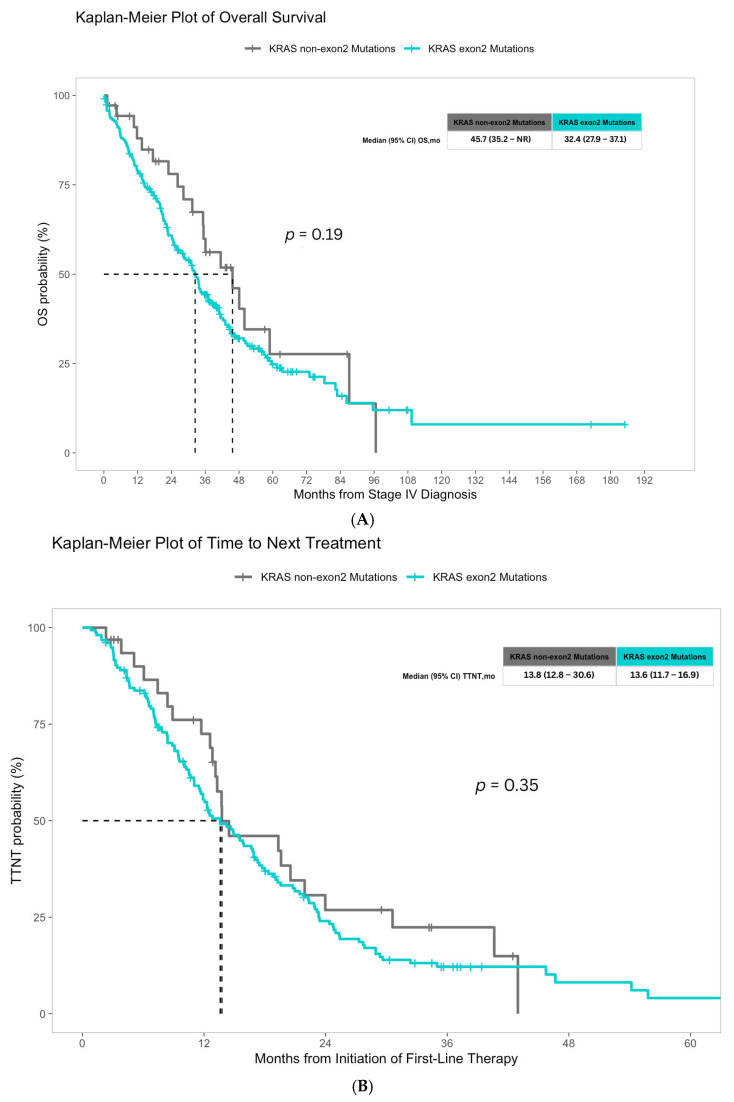
Kaplan-Meier survival curves for overall survival (OS) and time to next treatment (TTNT) in KRAS exon 2 and non-exon 2 mutated microsatellite stable metastatic colorectal cancer. (**A**) Overall survival and (**B**) Time to next treatment are shown for patients with KRAS non-exon 2 (*n* = 36) and exon 2 (*n* = 236) mutations. Median OS was 45.7 months in the non-exon 2 group and 32.4 months in the exon 2 group (log-rank *p* = 0.19). Median TTNT was 13.8 months in the non-exon 2 group and 13.6 months in the exon 2 group (log-rank *p* = 0.35). Dashed line indicates the 50th percentile (median) for each group.

All 272 patients were included in the survival analysis. At the time of data cutoff, 20 patients (56%) in the non-exon 2 group and 164 patients (69%) in the exon 2 group were deceased. Median OS was 45.7 months in the non-exon 2 group and 32.4 months in the exon 2 group. KRAS mutation subtype was not significantly associated with OS on univariable Cox regression analysis (HR 1.36, 95% CI 0.85–2.16; *p* = 0.2) (Figure 1).

For TTNT analysis, 32 non-exon 2 and 155 exon 2 patients with available first-line systemic treatment data were included. Among these, 23 (72%) non-exon 2 and 130 (84%) exon 2 patients progressed on first-line therapy and initiated second-line treatment. Median TTNT was 13.8 months in the non-exon 2 group and 13.6 months in the exon 2 group without statistically significant difference (HR 1.24, 95% CI 0.79-1.93; *p* = 0.3) (Figure 1).

### 3.5. The Impact of Inflammatory Markers on Overall Survival

#### 3.5.1. Exon 2 Mutations

**Figure 2:**
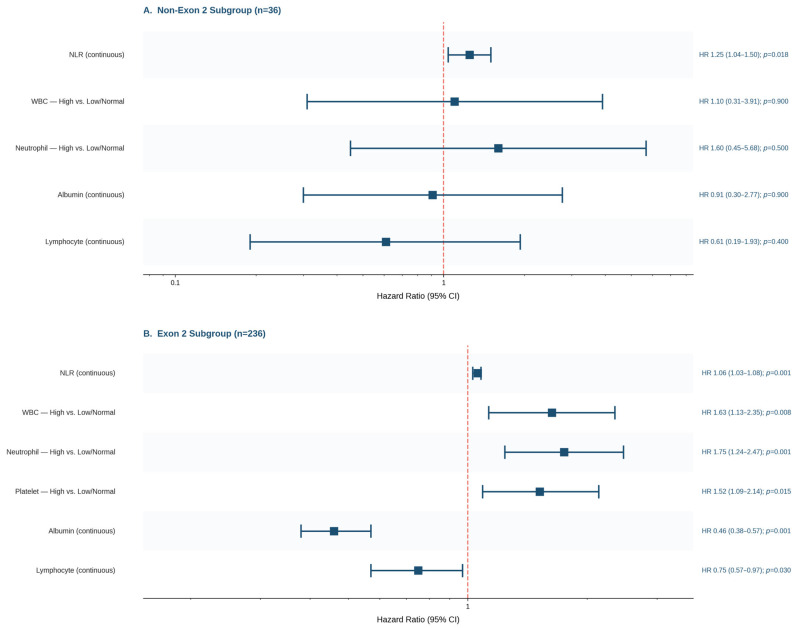
Association of systemic inflammatory markers with overall survival by KRAS mutation subtype. (**A**) Non-exon 2 subgroup (*n* = 36) and (**B**) exon 2 subgroup (*n* = 236). Hazard ratios (HR) and 95% confidence intervals (CI) were estimated using univariable Cox proportional hazards regression. The dashed vertical line represents HR = 1 (no effect). Categorical variables were analyzed as High vs. Low/Normal; continuous variables were analyzed per unit increase. PLR and platelet count were excluded from the forest plot as their per-unit hazard ratios approximated 1.00 upon rounding, despite reaching statistical significance; *p*-values for these variables are reported in the text.

In the exon 2 group, elevated WBC (HR 1.63, 95% CI 1.13–2.35; *p* = 0.008), elevated ANC (HR 1.75, 95% CI 1.24–2.47; *p* = 0.001), and elevated platelet count (HR 1.52, 95% CI 1.09–2.14; *p* = 0.015) were each associated with worse OS on categorical analysis. When analyzed as continuous variables, higher WBC (HR 1.11, 95% CI 1.06–1.16; *p* < 0.001), higher NLR (HR 1.06, 95% CI 1.03–1.08; *p* < 0.001), higher PLR (*p* < 0.001), higher ANC (HR 1.12, 95% CI 1.07–1.17; *p* < 0.001), and higher platelet count (*p* = 0.02) were similarly associated with worse OS. Lower albumin levels (HR 0.46, 95% CI 0.38–0.57; *p* < 0.001) were also associated with worse OS. Higher ALC as a continuous variable was associated with improved OS (HR 0.75, 95% CI 0.57–0.97; *p* = 0.03); categorical analysis was not performed due to insufficient events in the high lymphocyte group. These findings are summarized in Figure 2.

#### 3.5.2. Non-Exon 2 Mutations

In the non-exon 2 group (*n* = 36), NLR was the only inflammatory marker significantly associated with OS on univariable analysis (HR 1.25, 95% CI 1.04–1.50; *p* = 0.018). No other laboratory markers had significant association with OS (Figure 2).

#### 3.5.3. Multivariable Cox Regression Analysis

A multivariable Cox proportional hazards regression analysis was performed in the entire cohort to evaluate whether KRAS mutation subtype remained independently associated with overall survival after adjusting for clinicopathological and inflammatory confounders. Of 272 patients, 216 were included in the multivariable analysis after excluding those with missing covariate data.

On multivariable analysis, KRAS mutation subtype was not independently associated with OS (HR 1.376, 95% CI 0.794–2.383; *p* = 0.255), consistent with univariable findings. ECOG 2 (HR 2.797, 95% CI 1.527–5.123; *p* < 0.001) and multiple metastatic sites (HR 2.203, 95% CI 1.528–3.177; *p* < 0.001) were independent predictors of worse OS in the overall cohort. The multivariable analysis results are provided in Supplementary Table S1.

## 4. Discussion

Our study provides a comparative analysis of clinical, molecular, and inflammatory characteristics and their prognostic implications in KRAS exon 2 and non-exon 2 mutated MSS mCRC with several novel findings. We demonstrated that non-exon 2 KRAS-mutated CRC is more frequently observed in females, and primary tumors harboring non-exon 2 mutations are more commonly found in the left colon. Additionally, patients with exon 2 mutations had higher baseline platelet counts compared to the non-exon 2 group. Despite these clinicopathologic differences, KRAS mutation subtype did not serve as an independent prognostic factor on univariable or multivariable analysis (HR 1.376, 95% CI 0.794–2.383; *p* = 0.255) in our study. Notably, systemic inflammatory markers demonstrated differential prognostic significance across mutation subtypes. In the exon 2 group, inflammatory markers including elevated NLR, PLR, WBC, ANC, and low albumin predicted worse OS, whereas in the non-exon 2 group, only elevated NLR was associated with worse OS.

KRAS-mutated CRC is increasingly recognized as a heterogeneous disease [14]. Our current understanding of KRAS-related disease is primarily derived from exon 2 mutations, while data on non-exon 2 remain limited. This distinction is gaining clinical importance as the therapeutic landscape evolves. With the approval of sotorasib and adagrasib for the G12C variant [15,16], and ongoing trials targeting other KRAS mutations [17], characterizing individual mutation subtypes is becoming essential for patient selection and risk stratification.

In our cohort, the median OS was 45.7 months in the non-exon 2 group versus 32.4 months in the exon 2 group. While this 13.3-month difference is clinically notable, it did not reach statistical significance likely due to the cohort size. Literature regarding survival in these subgroups remains inconsistent. Ikoma et al. demonstrated a significant survival advantage for the exon 2 group [18], whereas Kojitani et al. reported no significant difference, though their minor RAS group similarly trended toward worse OS (exon 2 36.6 months vs. minor RAS 23.9 months; HR 0.95, 95% CI 0.55–1.65; *p* = 0.85) [19]. In contrast, our non-exon 2 cohort trended toward longer OS. These discrepancies likely stem from differences in patient selection. While both Japanese studies included MSI-H tumors, our cohort was restricted to MSS disease which represents a more homogenous group due to the fact that MSI-H status is independently prognostic for survival outcomes. Furthermore, Kojitani et al. included NRAS mutations in their analysis, whereas our study focused exclusively on KRAS mutation subtypes. Differences in racial composition may have further contributed, as both Japanese cohorts enrolled predominantly Asian patients whereas our cohort was primarily White. This difference in racial composition is relevant, as ethnic background can influence the frequency and biological behavior of KRAS mutations [20]. Adding to this complexity, Guo et al. examined prognostic outcomes across non-exon 2 subtypes, finding that exon 3 mutations were associated with the poorest prognosis, while exon 4 mutations showed the most favorable outcomes, approaching those of KRAS wild-type tumors [21]. However, their analysis included patients across all disease stages, limiting direct comparability with our cohort, which focuses only on stage IV disease. Collectively, these findings highlight the need for larger, stage-stratified studies to independently examine each KRAS mutation subtype and characterize their prognostic implications.

Notably, our survival results reflect a real-world perspective. Clinical trials often focus on patients with higher performance status due to their strict eligibility criteria. In contrast, our cohort included the full clinical spectrum, including those who were deemed unfit for systemic therapy and referred directly to hospice at diagnosis. While this broad inclusion leads to greater variability, it also provides a more accurate representation of routine clinical practice.

A key and novel finding of our study was the differential prognostic significance of systemic inflammatory markers across KRAS subtypes. In the exon 2 group, a broad range of markers, including elevated NLR, PLR, WBC, ANC, and low serum albumin (a negative acute phase reactant reflecting systemic inflammation [22]), were significantly associated with worse OS. In contrast, only NLR remained prognostic in the non-exon 2 group. The prognostic value of NLR and PLR is well established in CRC [8,23], and our findings extend this observation to the context of KRAS mutation subtypes. It is important to note that simultaneously testing multiple inflammatory markers raises the risk of type I error. While multiple comparison corrections would address this concern, applying them to an already limited sample, particularly in the non-exon 2 subgroup, would have further reduced statistical power and risked missing true associations. These analyses were therefore conducted without correction, consistent with their exploratory nature, and findings should be interpreted accordingly. With this caveat acknowledged, the differential prognostic significance of inflammatory markers across KRAS subtypes may reflect underlying differences in the tumor immune microenvironment. KRAS has been implicated as an active modulator of the tumor immune microenvironment, with distinct immune infiltration patterns reported across mutation subtypes [9]. This suggests that the more pronounced prognostic role of inflammatory markers in exon 2 disease may, in part, be a consequence of the distinct immune landscape driven by mutation subtypes. While biologically plausible, this mechanistic explanation remains speculative in our cohort as immune profiling and cytokine measurements were not available given the retrospective study design. Larger, prospective studies incorporating routine specimen collection and immune profiling will be needed to validate this hypothesis and provide sufficient statistical power for formal multiple comparison correction.

The significant association between non-exon 2 mutations and left-sided tumor location aligns with prior reports [19]. As previously noted, Kojitani et al. included NRAS mutations within their minor RAS group, and since NRAS mutations are independently more prevalent in left-sided CRC [24], this may have influenced their findings. Notably, left-sided predominance was observed across our entire cohort, likely attributable to our MSS-only inclusion criteria, as MSI-H tumors are disproportionately right-sided [25]. Whether this enrichment of non-exon 2 mutations reflects distinct oncogenic mechanisms or is a consequence of MSS-restricted biology remains unclear. Nevertheless, recognizing these anatomical patterns is important for a more granular understanding of KRAS-mutated CRC, particularly given the established prognostic significance of tumor sidedness in mCRC [26].

The female predominance in our non-exon 2 group is an intriguing finding. Prior studies have reported inconsistent findings regarding sex distribution across KRAS mutation subtypes. Guo et al. found that both exon 2 and exon 3 mutations were independently associated with female sex [21], whereas Kojitani et al. found no significant sex difference between KRAS exon 2 and minor RAS mutations [19]. As with survival data, direct comparisons are limited by the heterogeneous composition of existing cohorts. While sex-specific variations in oncogenic signaling [27] and the immune microenvironment [28] may contribute to these patterns, the biological basis for our observation remains to be fully defined. Our findings point toward a potential link between sex and molecular phenotype that remains underexplored in our current understanding of KRAS-mutant mCRC.

Our study has several limitations that warrant consideration. First, retrospective design introduces inherent selection bias and limits causal inference. Second, the non-exon 2 group was relatively small (*n* = 36), which may have limited statistical power to detect survival differences. The absence of statistically significant findings in this group should be interpreted with caution, as it may reflect limited statistical power rather than a true absence of effect. Third, inflammatory markers were assessed at a single time point, at the time of stage IV diagnosis, and do not capture dynamic changes throughout the disease course. Fourth, our cohort was predominantly White and derived from a single institution, limiting generalizability to more diverse populations. Fifth, given the exploratory and hypothesis-generating nature of this study, correction for multiple comparisons was not applied to the inflammatory marker analyses. Findings should be interpreted with this in mind, and confirmation in future studies remains necessary before definitive conclusions can be drawn. Sixth, non-exon 2 KRAS mutations were analyzed as a single group due to the limited number of cases, which precluded subgroup analyses at the individual exon level. However, exon 3 and exon 4 mutations may carry different biological and prognostic implications that could not be captured in the current analysis. Finally, TTNT was used as a surrogate for progression-free survival; however, this measure may be influenced by non-biological factors such as physician practice patterns, treatment-related toxicity, and patient preference, which could introduce bias into this analysis.

## 5. Conclusions

Our study provides insight into the distinct clinicopathologic and inflammatory landscapes of exon 2 versus non-exon 2 mutated MSS mCRC. Our findings suggest that systemic inflammation holds greater prognostic weight in exon 2 disease, whereas non-exon 2 subtypes may follow a different biological trajectory. As the therapeutic landscape for KRAS-mutated CRC continues to evolve, these findings hold promise for informing mutation-specific approaches to patient stratification and treatment planning. Prospective studies with larger, stratified cohorts are needed to validate these findings and delineate the distinct clinical profiles of individual KRAS mutation subtypes.

## Figures and Tables

**Table 1:** Baseline Clinical, Inflammatory and Molecular Characteristics.

		Non-Exon 2	Exon 2	*p*-Value
		(*n* = 36)	(*n* = 236)	
		% (n) or Median (Min–Max)	% (n) or Median (Min–Max)	
**Demographic characteristics**				
**Age at initial diagnosis**		61.0 (28–86)	61.5 (34–96)	0.255
Sex	Female	64% (23)	45% (106)	**0.048**
	Male	36% (13)	55% (130)	
Race	White	89% (32)	83% (197)	0.562
	Black or African American	8.3% (3)	13% (30)	
	Hispanic or Latino	0%	0.4% (1)	
	Asian	2.8% (1)	1.7% (4)	
	Not disclosed	0%	1.7% (4)	
Smoking Status	Never	64% (23)	52% (122)	0.461
	Current	8.3% (3)	13% (30)	
	Former	28% (10)	36% (84)	
ECOG Performance Status	ECOG-0	64% (18)	46% (94)	0.12
	ECOG-1	36% (10)	39% (80)	
	ECOG-2	0%	12% (24)	
	ECOG-3	0%	2.9% (6)	
**Tumor characteristics**				
Tumor Location	Colon	69% (25)	72% (171)	0.694
	Rectum	31% (11)	28% (65)	
Tumor Sidedness	Left	83% (30)	64% (152)	**0.035**
	Right	17% (6)	35% (83)	
	Unknown	0%	0.4% (1)	
**Metastatic sites**				
Metastatic Burden	Single metastatic site	56% (20)	61% (144)	0.584
	Multiple metastatic sites	44% (16)	39% (91)	
Liver Metastasis	Yes	72% (26)	62% (147)	0.271
	No	28% (10)	38% (89)	
Lung Metastasis	Yes	36% (13)	39% (93)	0.855
	No	64% (23)	61% (143)	
Peritoneal Metastasis	Yes	19% (7)	22% (53)	0.830
	No	81% (29)	78% (183)	
Other Metastasis	Yes	33% (12)	27% (64)	0.432
	No	67% (24)	73% (172)	
**First-line treatment**				
Delivery of Treatment	Yes	89% (32)	66% (155)	**0.004**
	No	11% (4)	34% (81)	
First-Line Regimen	Oxaliplatin-based doublet	42% (15)	41% (97)	**0.003**
	Irinotecan-based doublet	36% (13)	14% (32)	
	Triplet	8.3% (3)	4.7% (11)	
	Fluoropyrimidine monotherapy	0%	3.8% (9)	
	Other	2.8% (1)	2.5% (6)	
	No treatment	11% (4)	34% (81)	
**Inflammatory markers**				
**WBC Count (×10^3^/μL)**		7.05 (3.1–12.2)	7.90 (2.8–25.0)	**0.032**
WBC Interpretation	High	11% (4)	23% (55)	0.179
	Low/Normal	78% (28)	74% (174)	
	Unknown	11% (4)	3.0% (7)	
**ANC (×10^3^/μL)**		5.20 (1.79–11.3)	5.66 (1.4–22.0)	0.205
ANC Interpretation	High	11% (4)	26% (62)	0.123
	Low/Normal	75% (27)	69% (163)	
	Unknown	14% (5)	4.7% (11)	
**ALC (×10^3^/μL)**		1.20 (0.2–2.5)	1.30 (0.2–4.2)	0.071
ALC Interpretation	High	0%	0.8% (2)	1
	Low/Normal	83% (30)	94% (222)	
	Unknown	17% (6)	5.1% (12)	
**Platelet Count (×10^3^/μL)**		257 (90–518)	284 (73–833)	0.374
Platelet Interpretation	High	5.6% (2)	28% (65)	**0.008**
	Low/Normal	81% (29)	69% (164)	
	Unknown	14% (5)	3.0% (7)	
**NLR**		4.31 (2.06–17.0)	3.91 (0.47–90.5)	0.45
**PLR**		230.91 (122.4–2040)	208.39 (42.94–1630)	0.43
**Albumin (g/dL)**		3.85 (2.5–4.4)	3.70 (0.6–4.8)	0.177
**Molecular characteristics**				
**PIK3CA co-mutation**	Yes	8.3% (3)	11% (27)	0.778
	No	92% (33)	89% (209)	
**TMB (mut/Mb)**		9.36 (1.0–26.03)	8.86 (0.0–38.75)	0.546

Abbreviations: NLR, neutrophil-to-lymphocyte ratio; PLR, platelet-to-lymphocyte ratio; TMB, tumor mutational burden; ECOG, Eastern Cooperative Oncology Group; WBC, white blood cell count; ANC, absolute neutrophil count; ALC, absolute lymphocyte count. *p*-values were calculated using Fisher’s Exact test for categorical variables and Mann-Whitney U test for continuous variables. A two-sided *p*-value of <0.05 was considered statistically significant. Statistically significant *p*-values are shown in bold.

## Data Availability

The data that support the findings of this study are available from the corresponding author upon reasonable request.

## Supplementary Materials

The following supporting information can be downloaded at: https://www.mdpi.com/article/10.3390/cancers18132158/s1, Supplementary Table S1. Multivariable Cox Proportional Hazards Regression Analysis.

## Abbreviations

The following abbreviations are used in this manuscript:

ALC	absolute lymphocyte count
ANC	absolute neutrophil count
CEA	carcinoembryonic antigen
CI	confidence interval
CRC	colorectal cancer
ECOG	Eastern Cooperative Oncology Group
EGFR	epidermal growth factor receptor
HR	hazard ratio
IRB	Institutional Review Board
KRAS	Kirsten rat sarcoma
mCRC	metastatic colorectal cancer
MSI	microsatellite instability
MSS	microsatellite stable
NGS	next-generation sequencing
NLR	neutrophil-to-lymphocyte ratio
NRAS	neuroblastoma RAS viral oncogene
OS	overall survival
PLR	platelet-to-lymphocyte ratio
RAS	rat sarcoma
TMB	tumor mutational burden
TTNT	time to next treatment
UPMC	University of Pittsburgh Medical Center
WBC	white blood cell count
