# Supplementary material for: A Clinical and Molecular Comparative Analysis of KRAS Exon 2 and KRAS Non-Exon 2 Mutated Colorectal Cancer
Source: Cancers (Basel). 2026 Jul 5;18(13):2158. doi: 10.3390/cancers18132158 (PMC13359703; doi:10.3390/cancers18132158)
Supplement: Supplementary file 1 [file cancers-18-02158-s001.zip › cancers-4370588-supplementary.pdf]

Supplementary Table S1. Multivariable Cox Proportional Hazards Regression Analysis

Variable	HR	95% CI	p-value
**KRAS MUTATION SUBTYPE**			
KRAS Exon 2 vs. Non-Exon 2	1.376	0.794–2.383	0.255
**CLINICAL CHARACTERISTICS**			
Age at diagnosis	1.000	0.985–1.016	0.970
ECOG 1	1.129	0.794–1.605	0.500
ECOG 2	2.797	1.527–5.123	**<0.001**
ECOG 3	2.057	0.742–5.705	0.166
Left-sided tumor	1.019	0.711–1.461	0.919
Delivery of systemic treatment	0.744	0.502–1.102	0.140
Multiple metastatic sites	2.203	1.528–3.177	**<0.001**
PIK3CA co-mutation	1.006	0.598–1.692	0.983
**INFLAMMATORY MARKERS**			
PLR (per unit)	1.002	1.000–1.003	0.051
NLR (per unit)	0.985	0.939–1.034	0.550
Albumin (per unit)	0.610	0.451–0.825	**0.001**

Multivariable Cox proportional hazards regression was performed in the overall cohort including KRAS mutation subtype, age at diagnosis, ECOG performance status, tumor sidedness, delivery of first-line systemic treatment, metastatic burden (single vs. multiple sites of metastasis), PIK3CA co-mutation status, PLR, NLR, and serum albumin as covariates. Reference categories: KRAS non-exon 2, ECOG 0, right-sided tumor, no treatment. HR, hazard ratio; CI, confidence interval; PLR, platelet-to-lymphocyte ratio; NLR, neutrophil-to-lymphocyte ratio; ECOG, Eastern Cooperative Oncology Group. Significant p-values (<0.05) are shown in bold.
